# Computational Models for Prediction of Yeast Strain Potential for Winemaking from Phenotypic Profiles

**DOI:** 10.1371/journal.pone.0066523

**Published:** 2013-07-16

**Authors:** Inês Mendes, Ricardo Franco-Duarte, Lan Umek, Elza Fonseca, João Drumonde-Neves, Sylvie Dequin, Blaz Zupan, Dorit Schuller

**Affiliations:** 1 CBMA (Centre of Molecular and Environmental Biology)/Department of Biology/University of Minho, Braga, Portugal; 2 Faculty of Administration, University of Ljubljana, Ljubljana, Slovenia; 3 Faculty of Computer and Information Science, University of Ljubljana, Ljubljana, Slovenia; 4 Research Center for Agricultural Technology – Department of Agricultural Sciences, University of Azores, Ponta Delgada, São Miguel, Azores, Portugal; 5 INRA (Institut National de la Recherche), UMR1083, Sciences pour l'Enologie, Montpellier, France; University of Strasbourg, France

## Abstract

*Saccharomyces cerevisiae* strains from diverse natural habitats harbour a vast amount of phenotypic diversity, driven by interactions between yeast and the respective environment. In grape juice fermentations, strains are exposed to a wide array of biotic and abiotic stressors, which may lead to strain selection and generate naturally arising strain diversity. Certain phenotypes are of particular interest for the winemaking industry and could be identified by screening of large number of different strains. The objective of the present work was to use data mining approaches to identify those phenotypic tests that are most useful to predict a strain's potential for winemaking. We have constituted a *S. cerevisiae* collection comprising 172 strains of worldwide geographical origins or technological applications. Their phenotype was screened by considering 30 physiological traits that are important from an oenological point of view. Growth in the presence of potassium bisulphite, growth at 40°C, and resistance to ethanol were mostly contributing to strain variability, as shown by the principal component analysis. In the hierarchical clustering of phenotypic profiles the strains isolated from the same wines and vineyards were scattered throughout all clusters, whereas commercial winemaking strains tended to co-cluster. Mann-Whitney test revealed significant associations between phenotypic results and strain's technological application or origin. Naïve Bayesian classifier identified 3 of the 30 phenotypic tests of growth in iprodion (0.05 mg/mL), cycloheximide (0.1 *µ*g/mL) and potassium bisulphite (150 mg/mL) that provided most information for the assignment of a strain to the group of commercial strains. The probability of a strain to be assigned to this group was 27% using the entire phenotypic profile and increased to 95%, when only results from the three tests were considered. Results show the usefulness of computational approaches to simplify strain selection procedures.

## Introduction

Most European wine producers use commercial starter yeasts to guarantee the reproducibility and the predictability of wine quality. The advantages of fermentations containing *Saccharomyces cerevisiae* starter cultures relies on the fact that they are rapid and produce wine with desirable organoleptic characteristics through successive processes and harvests [1], [2]. In these fermentations the winemaker has control over the microbiology of the process, because it is expected that the inoculated yeast strain predominates and suppresses the indigenous flora. Currently, there are about 200 commercial *S. cerevisiae* winemaking strains available, and it is a common practice among wineries to use commercial starter yeasts that were obtained in other winemaking regions.

*S. cerevisiae* strains from diverse natural habitats harbour a vast amount of phenotypic diversity [3], driven by interactions between yeast and the respective environment. In grape juice fermentations, strains are exposed to a wide array of biotic and abiotic stressors [4], which may lead to strain selection and generate naturally arising strain diversity. Outside the wineries, this diversifying selection occurs due to unique pressures imposed after expansion into new habitats [5]–[9]. This agrees with findings showing that wine and sake strains are phenotypically more diverse than would be expected from their genetic relatedness [10].

Recent phylogenetic analyses of *S. cerevisiae* strains showed that the species as a whole consists of both “domesticated” and “wild” populations. DNA sequence analysis revealed that domesticated strains derived from two independent clades, corresponding to strains from winemaking and sake. “Wild” populations are mostly associated with oak trees, nectars or insects [11]–[13]. Although some *S. cerevisiae* strains are specialized for the production of alcoholic beverages, they were derived from natural populations that were not associated with industrial fermentations. This was proposed once that the oldest lineages and the majority of variation were found in strains from sources unrelated to wine production [14].

The phenotypic diversity of *S. cerevisiae* strains has been explored for decades in strain selection programmes to choose the ones that enhance the wine's sensorial characteristics and confer typical attributes to specific wines. These strains are used as commercial ones by winemakers to efficiently ferment grape musts and produce desirable metabolites, associated with reduced off-flavours [15], [16]. Strain selection approaches are mentioned in many studies aiming to characterize *S. cerevisiae* isolates obtained from winemaking regions worldwide. The most relevant physiological tests refer to fermentation rate and optimum fermentation temperature, stress resistance (ethanol, osmotic and acidic), killer phenotype, sulphur dioxide (SO_2_) tolerance and production, hydrogen sulphide (H_2_S) production, glycerol and acetic acid production, synthesis of higher alcohols (e.g. isoamyl alcohol, n-propanol, isobutanol), *β-*galactosidase and proteolytic enzyme activity, copper resistance, foam production and flocculation [17].

In our previous work [18] we evaluated the phenotypic and genetic variability of 103 *S. cerevisiae* strains from the *Vinho Verde* wine region (Northwest Portugal). We then applied several data mining procedures to estimate a strain's phenotypic behaviour based on its genotypic data. We used mainly taxonomic tests and strains from winemaking environments of one geographical origin. This study was, to our best knowledge, the first attempt to computationally associate genotypic and phenotypic data of *S. cerevisiae* strains. We used subgroup discovery techniques to successfully identify strains with similar genetic characteristics (microsatellite alleles) that exhibited similar phenotypes.

Within the present study we expanded the strain collection to 172 isolates from worldwide geographical origins and technological groups (wine, bread, sake, etc.) and included 30 tests with biotechnological relevance for the selection of winemaking strains.

Our objective was to gain a deeper understanding of the phenotypic diversity of a global strain collection and to infer computational models that predict the biotechnological potential or geographic origin of a strain from its phenotypic profile.

## Results

### Phenotypic characterization of the strain collection

**Figure 1:**
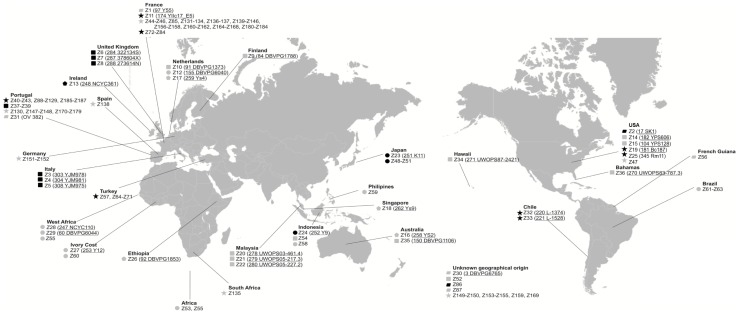
Geographical location of 172 yeast strains. Underlined identifiers indicate the original designation of sequenced strains [12]. Symbols represents the strains technological applications or origin: black star – wine and vine; grey star – commercial wine strain; black square – clinical; grey square – natural isolates; black circle – sake; grey circle – other fermented beverages; black pentagon – beer; grey pentagon- baker; black rectangle – laboratory; grey rectangle – unknown biological origin.

A *Saccharomyces cerevisiae* collection was constituted with 172 strains obtained from different geographical origins as shown in the map in Figure 1. As detailed in Table S1 (supplementary data), the technological applications or environments from where the strains were derived were: wine and vine (74 isolates), commercial wine strains (47 isolates), other fermented beverages (12 isolates), other natural environments – soil woodland, plants and insects (12 isolates), clinical (9 isolates), sake (6 isolates), bread (4 isolates), laboratory (3 isolates), beer (1 isolate), and four isolates with unknown origin.

A phenotypic screen was devised to evaluate strain-specific patterns for a set of physiological tests, including also tests that are important for winemaking strain selection. The first group of tests were performed in microplates using supplemented grape must, whereas a high reproducibility was obtained between experimental replicates. The second set of tests consisted in the evaluation of growth in solid culture media (BiGGY medium, Malt Extract Agar supplemented with ethanol and sodium metabisulfite). Galactosidase activity was evaluated by growth evaluation using Yeast Nitrogen Base supplemented with galactose, as indicated in the materials and methods section. After incubation, growth was evaluated by visual scoring (solid media) or by A_640_ determination (liquid media). Table 1 summarizes the number of strains belonging to each of the phenotypic classes. Similarities between strains were evident, but each strain showed a unique phenotypic profile.

**Table 1:** Number of strains belonging to different phenotypic classes, regarding values of optical density (Class 0: A_640_ = 0.1; Class 1: 0.2<A_640_>0.4; Class 2: 0.5<A_640_>1.0; Class 3: A_640_>1.0), growth patterns in solid media, or colour change in BiGGY medium.

Phenotypic test	Type of medium	Phenotypic class of growth			
		0	1	2	3
30°C	liquid (must)	0	0	3	168
18°C	liquid (must)	51	120	1	0
40°C	liquid (must)	28	14	80	50
pH 2	liquid (must)	101	68	3	0
pH 8	liquid (must)	0	0	19	153
KCl (0.75 M)	liquid (must)	0	2	146	24
NaCl (1.5 M)	liquid (must)	84	79	9	0
CuSO_4_ (5 mM)	liquid (must)	124	45	3	0
SDS (0.01% w/v)	liquid (must)	139	32	1	0
Ethanol 6% (v/v)	liquid (must)	0	2	36	134
Ethanol 10% (v/v)	liquid (must)	17	28	85	42
Ethanol 14% (v/v)	liquid (must)	82	35	50	5
Ethanol 12% (v/v)	solid (MEA)	150	20	1	1
Ethanol 12% (v/v) + Na_2_S_2_O_5_ (75 mg/L)	solid (MEA)	159	14	0	0
Ethanol 12% (v/v) + Na_2_S_2_O_5_ (100 mg/L)	solid (MEA)	169	3	0	0
Ethanol 14% (v/v) + Na_2_S_2_O_5_ (50 mg/L)	solid (MEA)	148	24	0	0
Ethanol 16% (v/v) + Na_2_S_2_O_5_ (50 mg/L)	solid (MEA)	163	9	0	0
Ethanol 18% (v/v) + Na_2_S_2_O_5_ (50 mg/L)	solid (MEA)	165	7	0	0
KHSO_3_ (150 mg/L)	liquid (must)	34	11	26	101
KHSO_3_ (300 mg/L)	liquid (must)	57	19	29	67
Wine supplemented with glucose (0.5% w/v)	liquid	103	45	24	0
Wine supplemented with glucose (1% w/v)	liquid	115	41	16	0
Iprodion (0.05 mg/mL)	liquid (must)	1	0	28	143
Iprodion (0.1 mg/mL)	liquid (must)	1	1	13	157
Procymidon (0.05 mg/mL)	liquid (must)	0	0	7	165
Procymidon (0.1 mg/mL)	liquid (must)	1	0	9	162
Cycloheximide (0.05 *µ*g/mL)	liquid (must)	3	0	7	162
Cycloheximide (0.1 *µ*g/mL)	liquid (must)	2	1	19	150
H_2_S production	solid (BiGGY)	1	11	105	55
Galactosidase activity	liquid (YNB)	0	21	98	53

MEA: Malt Extract Agar.

A total of 5160 phenotypic data points were obtained, from 172 strains and 30 tests. The concentrations of the added compounds were chosen to obtain a wide range of tolerance patterns. As expected, all strains grew well at 30°C, contrary to the growth at 40°C, where a large phenotypic diversity was observed. Most strains were able to grow well at pH 8, contrarily to the pH value of 2. As expected, cellular growth decreased with increasing concentrations of ethanol (6–14% v/v, liquid media), whereas only five isolates were able to grow well at the highest ethanol concentration of 14% (v/v). When ethanol was combined with sodium metabisulfite in solid culture media, growth was reduced with increasing concentrations of ethanol (12 to 18%, v/v) or sodium metabisulfite (50–100 mg/L). Resistance to sulphur dioxide, which is an antioxidant and bacteriostatic agent used in vinification, was tested by growth in the presence of wine must supplemented with potassium bisulphite (KHSO_3_). For the concentrations of 150 and 300 mg/L, 101 and 67 strains achieved the highest class of growth, respectively. Resistance to the fungicides iprodion, procymidon and to cycloheximide was rather high at the indicated concentrations. Hydrogen sulphide production was tested using BiGGY medium. The majority of the strains were intermediate H_2_S producers with the exception of one strain (from the group of wine and vine strains) that did not produce H_2_S.

**Figure 2:**
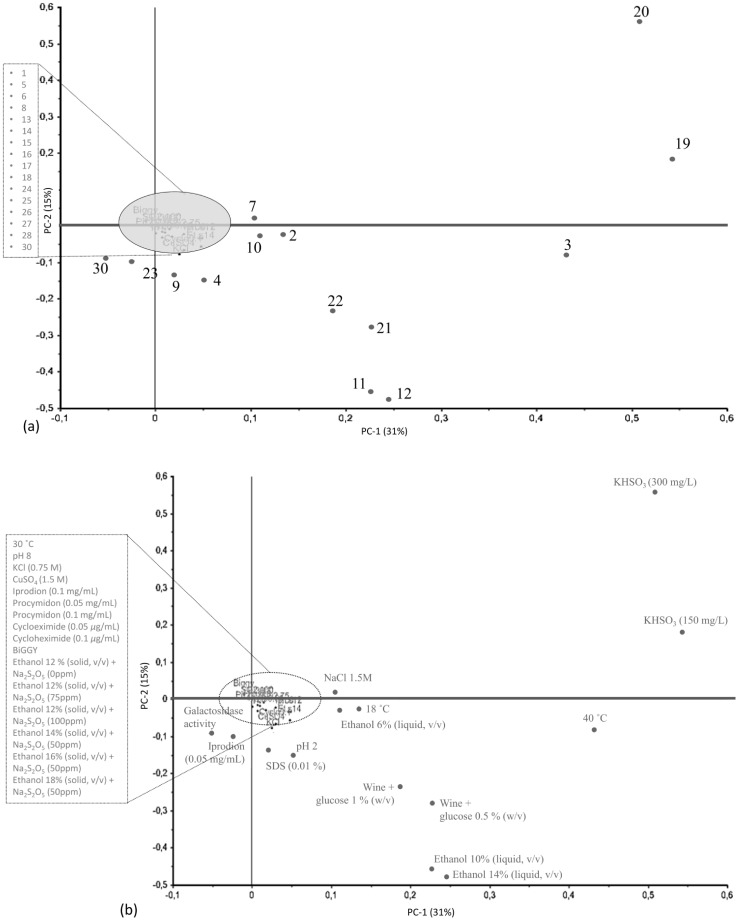
Principal component analysis of phenotypic data for 172 strains. (**a**) −30 phenotypic tests (loadings). Numbers indicate phenotypic tests, as mentioned in Table 1: (1) −30°C; (2) −18°C; (3) −40°C; (4) – pH 2; (5) – pH 8; (6) – KCl (0.75 M); (7) – NaCl (1.5 M); (8) – CuSO_4_ (1.5 M); (9) – SDS (0.01%); (10) – ethanol 6% (v/v) liquid medium; (11) – ethanol 10% (v/v) liquid medium; (12) – ethanol 14% (v/v) liquid medium; (13) – ethanol 12% (v/v) solid medium; (14) – ethanol 12% (v/v) solid medium + Na_2_S_2_O_5_ (75 mg/L); (15) – ethanol 12% (v/v) solid medium + Na_2_S_2_O_5_ (100 mg/L); (16) – ethanol 14% (v/v) solid medium + Na_2_S_2_O_5_ (50 mg/L); (17) – ethanol 16% (v/v) solid medium + Na_2_S_2_O_5_ (50 mg/L); (18) – ethanol 18% (v/v) solid medium + Na_2_S_2_O_5_ (50 mg/L); (19) – KHSO_3_ (150 mg/L); (20) – KHSO_3_ (300 mg/L); (21) – wine supplemented with glucose 0.5% (w/v); (22) – wine supplemented with glucose 1% (w/v); (23) – Iprodion (0.05 mg/mL); (24) – Iprodion (0.1 mg/mL); (25) – Procymidon (0.05 mg/mL); (26) –Procymidon (0.1 mg/mL); (27) – Cycloheximide (0.05 *µ*g/mL); (28) – Cycloheximide (0.1 *µ*g/mL); (29) – H_2_S production; (30)– galactosidase activity. (**b**) – 172 strains (scores) distribution. Symbols represents the strains technological applications or origin: black star – wine and vine; grey star – commercial wine strain; black square – clinical; grey square – natural isolates; black circle – sake; grey circle – other fermented beverages; black pentagon – beer; grey pentagon- baker; black rectangle – laboratory; grey rectangle – unknown biological origin.

A global view of strain's phenotypic diversity is shown in Figures 2 and S1. Principal component analysis (PCA) of phenotypic data (Figure 2) show the segregation of all 172 strains (scores) and the loadings for phenotypic variables in the first two PCA components. The phenotypes responsible for the highest strain variability (Figure 2a) were associated with growth patterns in the presence of potassium bisulphite (KHSO_3_), at 40°C, in a finished wine supplemented with glucose (0.5%, w/v), and resistance to ethanol in liquid media (10 and 14%, v/v). PC-1 (31%) and PC-2 (15%) explained 46% of strain variability and segregated strains by phenotypic behaviour into some patterns, as shown in Figure 2b. The group of sake strains (dark dot) and the group of natural strains (dark square), tended to be separated by the second component, accumulating in the lower part of the PCA, indicating that they were influenced by the presence of ethanol in the medium (higher resistance), and by the growth in the presence of potassium bisulphite (300 mg/L, lower resistance). Strains isolated from vines or wine (dark star) showed a heterogeneous phenotypic behaviour since they were dispersed throughout the PCA plot for both components. A similar tendency was observed for commercial strains (light star); however, the majority of strains tended to concentrate in the upper part of the PCA, indicative of a trend to higher KHSO_3_ resistance and lower ethanol resistance. The nine clinical strains were distributed in both PCA components, showing no discriminant results in any of the phenotypic tests.

UPGMA (Unweighted Pair Group Method with Arithmetic Mean) algorithm was used to hierarchical cluster the 172 strains. The dissimilarity between two strains was measured using Euclidean distance (Figure S1). The combined phenotypes of wine strains did not separate this group of strains that were rather scattered throughout all the clusters. Commercial strains (light star) tended to be more predominant in the clusters shown in the lower part of the dendogram, where some of the clusters are constituted only by commercial strains.

We further analysed phenotypic diversity through *k*-means clustering algorithm. Using silhouette score [19] we identified 3 distinct clusters (Table 2), composed of 38, 90 and 44 strains respectively. The phenotypes that most distinguished the strains, as indicated by high values of information gain to classify strains into clusters, were growth at the highest and lowest temperature tested (18 and 40°C). Cluster 2 was constituted of strains that didn't grow at both 18 and 40°C, whereas cluster 1 and 3 included strains that grew at both temperatures, but with more pronounced growth at 40°C, in particular for strains of cluster 3. Other tests that were also relevant for the cluster separation included growth in the presence of NaCl (1.5 M), KHSO_3_ (150 and 300 mg/L), ethanol 6% (v/v) and at pH 2. The strain cluster membership is displayed in the phenotypic data PCA visualization (supplementary Figure S2).

**Table 2:** Phenotypic tests mostly contributing for the division of strains into three clusters, in terms of information gain, obtained with *k*-means clustering algorithm.

Phenotypic test	Information gain	Cluster		
		1	2	3
18°C	0,33	1	0	1
40°C	0,33	2	0	3
NaCl (1.5M)	0,26	0	0	1
KHSO_3_ (300 mg/L)	0,23	3	0	3
Ethanol 6% (v/v) – liquid medium	0,23	3	2	3
pH 2	0,21	0	0	1
KHSO_3_ (150 mg/L)	0,21	3	0	3
**Total number of strains**		38	90	44

Numbers in the last three columns represent the most characteristic value in terms of phenotypic class of strains included in the clusters, for the mentioned phenotypic tests.

### Statistical analysis

The number of strains belonging to each group of technological applications or environment varies between 1 and 74. To assess a possible influence of a sample bias, due to an unequal number of representatives from each group, we determined the 95% confidence intervals for average Manhattan distance [20] between two strains in a selected group (composed by at least 5 strains). The distance was estimated based on the strain's entire phenotypic profile. The lower and upper bound of each confidence interval were determined by percentiles of average distances for 10000 bootstraps samples. For example, with this analysis we show that while the group of commercial strains (47 isolates) includes 31 commercial strains isolated in France, this should not bias our statistical analysis on utility of strains. Namely, the 95% confidence interval for average distances between pairwise combinations of commercial strains from France (6.37, 8.01) overlaps with the confidence interval of commercial strains from other geographical origins (4.97, 8.13). The inclusion of a high number of strains from France does not change the limits of the confidence interval of the group of commercial strains. A similar result was observed for the group of wine and vine strains that includes numerous strains from Portugal: the 95% confidence interval for average distances between pairwise combinations of strains from Portugal (8–12, 9.83) overlaps with the same interval for wine and vine strains from other geographical locations (8.06, 9.59).

Mann-Whitney test is mostly used to identify statistically significant associations between two data sets in which data instances in each group are measured on ordinal level and when there is an unequal number of members in the classes to be compared. This test was used to search for relationships between phenotypic results for the 172 strains, and their shared geographical origin or technological application group. After the dichotomization of variables (geographical origin and technological application or origin), Mann-Whitney test was performed for each phenotypic variable and *p*-values were computed and further adjusted using Bonferroni correction. Statistical analysis using Mann-Whitney test revealed 300 associations between phenotypes and technological application or origin of strains, whereas statistical significance was found for 11 associations (Bonferroni adjusted *p*-value lower than 0.1). For each phenotypic test, we computed the probability of each phenotypic class (0–3) according to its contribution to the observed association. The most significant associations between a phenotypic class and a technological group are reported in Table 3. Two associations were found for the resistance to iprodion, whereas class 3 and 2 were associated with strains collected from wine/vineyards and commercial strains, respectively. Capacity to grow in the presence of potassium bisulphite (150 mg/mL, classes 2 and 3) was associated with commercial wine strains. Natural isolates (87%–89%) were associated with class 2 of growth in wine supplemented with glucose, both at 0.5 and 1% (w/v), contrarily to 57% of commercial strains that were unable to grow in wine supplemented with glucose (0.5%, w/v). The lower ability of commercial strains to grow at higher ethanol concentrations was also supported by the finding of one significant association for absent growth (class 0) in liquid medium containing ethanol (14%, v/v). About half of the strains included in the groups shared the inability to grow in must containing SDS (0.01%, w/v) and CuSO_4_ (5 mM), but grew well in cycloheximide-supplemented must (76% of strains, class 2). An identical approach was undertaken to find associations between the phenotypic results and the geographical origin of strains, but no statistically relevant results were obtained (data not shown).

**Table 3:** Relevant associations (adjusted *p*<0.1) between phenotypic results and strain's technological application or origin, obtained using Mann-Whitney test and after Bonferroni correction.

Phenotypic test	Class of phenotypic result	Technological group/origin	Adjusted *p*-value	% of strains sharing positive association *
Iprodion (0.05 mg/mL)	2	Commercial	3.24×10^−8^	82.0
Iprodion (0.05 mg/mL)	3	Wine and vine	0.015	56.4
KHSO_3_ (150 mg/L)	2, 3	Commercial	0.001	59.3
Wine supplemented with glucose (0.5%, w/v)	0	Commercial	0.075	57.0
Wine supplemented with glucose (0.5%, w/v)	2	Natural isolate	0.002	87.2
Wine supplemented with glucose (1%, w/v)	2	Natural isolate	0.041	89.5
Ethanol 14% (v/v) – liquid medium	0	Commercial	0.004	64.5
Cycloheximide (0.1 *µ*g/mL)	2	Commercial	0.007	75.6
Procymidon (0.1 mg/mL)	2	Other fermented beverages	0.005	92.4
SDS (0.01%, w/v)	0	Commercial	0.078	45.3
CuSO_4_ (5 mM)	0	Commercial	0.075	50.6

* Percentage of strains that share the phenotypic result and belong to the described group or that didn't share the phenotypic result nor belong to that group.

### Prediction of technological group based on phenotypic results

Our next objective was to construct a model that would predict strain's technological group from its phenotypic profile. *K*-nearest neighbour algorithm (*k*NN) and naïve Bayesian classifiers [21], as implemented in the Orange data mining software were used for modelling.

**Figure 3:**
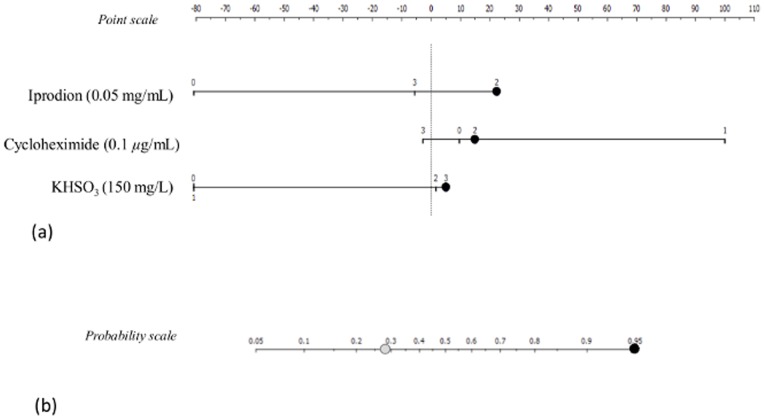
Nomogram showing naïve Bayesian classifier results for the prediction of commercial strains based on phenotypic classes of growth for each test. (**a**) Performance of three phenotypic tests that contributed in a positive way to predict commercial strains; (**b**) Probability of predicting commercial strains when considering the entire phenotypic profile (grey circle), or only the three phenotypic tests mentioned in panel (a) by the blue dots (black circle).

The predictive performance of both classifiers was evaluated in terms of area under the Receiver-Operating-Characteristics (ROC) curve, using 5-fold cross validation [22]. Table 4 shows the confusion matrix of naïve Bayesian classifications in test data sets of cross-validation; *k*NN results are not shown, as these were similar for both modelling techniques. Cross validated AUC score was 0.70. Correct assignments were found for the larger groups of commercial wine strains and strains obtained from wine and vineyards, where 36 (77%) and 54 (73%) strains respectively, were accurately allocated. The same computational technique was also used to explore which phenotypes mostly contributed to the assignment of a strain to the commercial wine group. Figure 3 represents a nomogram that shows naïve Bayesian classifier results [23]. Three phenotypes were considered by the classifier as the ones contributing more positively to build the model, having the remaining ones a smaller impact. To predict the commercial potential of a strain, the contribution of each phenotype was scored in the scale from −100 to 100, and the individual scores were summed-up to read-out the probability of the predicted class. For the present data set, growth in must containing the fungicide iprodion (0.05 mg/mL), in cycloheximide (0.1 *µ*g/mL) and in the presence of potassium bisulphite (150 mg/mL) were the three features with the most relevant contribution for the mathematical assignment of a strain to the commercial group (Figure 3a). The probability of a strain to be assigned to the group of commercial strains is 0.27 (27%) when considering the strains entire phenotypic profile and increases to 0.95 (95%) when only the three phenotypic results mentioned in Figure 3a are taken into consideration, as shown in the probability scale present in Figure 3b.

**Table 4:** Confusion matrix indicating the technological application or origin prediction of 172 strains and their predictions as obtained with naïve Bayesian classifier (AUC  = 0.70).

			Predicted technological application or origin									
		Total number of strains	Beer	Bread	Clinical	Commercial wine strain	Laboratory	Natural isolate	Other fermented beverages	Sake	Unknown biological origin	Wine and vine
**Real technological application or origin**	Beer	1	**0** (0%)	0	0	0	0	1	0	0	0	0
	Bread	4	0	**0** (0%)	0	0	0	3	0	0	0	1
	Clinical	9	0	0	**0** (0%)	2	0	1	0	0	1	5
	Commercial wine strain	47	0	0	3	**36** (77%)	0	2	1	0	0	5
	Laboratory	3	0	0	1	0	**0** (0%)	0	1	0	1	0
	Natural isolate	12	0	1	2	2	0	**2** (17%)	2	0	0	3
	Other fermented beverages	12	0	0	1	1	0	2	**3** (25%)	1	0	4
	Sake	6	0	0	0	0	0	1	1	**2** (33%)	0	2
	Unknown biological origin	4	0	0	1	0	0	0	1	0	**1** (25%)	1
	Wine and vine	74	0	1	3	8	1	2	3	1	1	**54** (73%)

## Discussion

Within our previous work [18] we developed computational techniques to relate the genotypes and phenotypes of 103 *Saccharomyces cerevisiae* strains from a winemaking region. The isolates were characterized regarding their allelic combinations for 11 microsatellites and phenotypic screens included mainly taxonomic criteria but also some tests with biotechnological relevance. Subgroups were found for strains sharing allelic combinations and specific phenotypes such as low ethanol resistance, growth at 30°C and growth in media containing galactose, raffinose or urea. Herein, we aim to extend the work to a phenotypically mostly heterogeneous strain collection of 172 *S. cerevisiae* isolates from worldwide origins, to computationally relate the phenotype with the strain's geographical origins and to make predictions about a strain's biotechnological potential based on phenotypic data. The group of phenotypic tests used herein was based on approaches that are generally applied for the selection of *S. cerevisiae* winemaking strains [17].

The collection of 172 strains from worldwide geographical origins revealed a high phenotypic diversity (Figures 2, S2 and Table 2), which is in agreement with previous studies [3], [10], [18], [24]–[27]. A significantly higher phenotypic diversity was observed in the present study compared to our results from 2009 using 103 Portuguese wine yeast strains [18]. In particular, the inclusion of new tests compared to our previous study allowed a more detailed analysis of the phenotypic variability of strains associated with winemaking environments. Recent studies aimed to describe the elements that shaped the genomes of *S. cerevisiae* strains, suggesting that populations comprise distinct domesticated and natural groups, as well as mosaics within these groups, based on the strain origin and application [12], [28], [29]. Clinical isolates for example, do not derive from a common ancestor, but rather represent multiple events in which environmental strains opportunistically colonize humans [28], [30].

Genetic rearrangements and intra-strain variation is characteristic for this species [31], [32], which might explain the rather high phenotypic variability that was described in recent studies. Camarasa [3] showed that some phenotypes (resistance to high sugar concentrations, ability to complete fermentation and low acetate production) were able to distinguish groups of strains according to their ecological niches, providing evidence for phenotypic evolution driven by environmental adaptation. This high phenotypic variation in stressful conditions was also revealed by Kvitek *et al*., showing the existence of unique features shared by strains from similar habitats [10]. Our data are in agreement with the previously mentioned studies regarding the high phenotypic diversity. They also confirm the findings of Legras and co-workers [33], that found populational substructures of *S. cerevisiae* strains according to their technological application or origin, using multilocus microsatellite typing. In the work of Legras only 28% of the diversity was associated with geographical origins, which suggests local domestication events. We herein investigated the utility of data mining to improve our understanding of relations between phenotypes and the strains technological application or origin. The developed models can also be useful to optimize screening tests and to find commercial wine yeast candidates from strain collections.

Using Mann-Whitney test, 11 significant associations were found between a particular phenotypic result and a technological application or origin of the strains (Table 3). The most significant results were found for the resistance to iprodion, growth in potassium bisulphite and in wine supplemented with glucose. Iprodion is a dicarboximide contact fungicide used to control a wide variety of fungal pests on vegetables, ornamentals, pome and stone fruit, root crops, cotton and sunflowers. *S. cerevisiae* shows a higher resistance to this fungicide than other yeast species such as *Candida albicans.* In this species iprodion stimulates glycerol synthesis and inhibits the cell growth for several days, contrarily to *S. cerevisiae* where a low toxicity was observed [34], [35]. Our results showed that iprodion resistance (0.05 mg/mL) was higher in strains from wine and vineyards compared to commercial wine strains. The higher iprodion resistance among strains obtained from wineries and vineyards might be explained by the evolution of this trait upon recurrent exposure, which does not apply for commercial wine strains that are added to clarified musts that should not contain this fungicide. The low ethanol resistance of commercial wine strains in liquid media containing 14% (v/v) ethanol was somehow unexpected, because these strains are usually selected for high ethanol resistance. This could be explained by the fact that the mathematical relations were observed for ethanol concentrations above the values that usually occur in wines (10–13%, v/v). Results showed also that commercial strains tended to a better growth in media containing potassium bisulphite, a compound used as wine antiseptic and antioxidant, reflecting also an adaptive mechanism among this group of strains.

We found that the large phenotypic variability between strains could be associated with the technological application or origin of the strains (Table 3) rather than their geographical origin, once that no relevant relations were considered for the last analysis. The naïve Bayesian classifier was used to assign a strain to their technological application or origin group, based on their phenotypic profile (Table 4). This association was achieved for the majority of strains belonging to the commercial and wine and vine groups (77% and 73% respectively). The cross-validated performance of this method yielded an AUC score of 0.70, that is considered as moderate [22] and lies in between the values of an arbitrary and perfect classification (AUC  = 0.5 and 1.0, respectively). Poor results were obtained for the remaining groups, which is due to the corresponding small number of isolates. These results demonstrate the potential of the predictive models to classify strains based on results of phenotypic screens.

Bayesian classifier used the strains phenotypic profiles for prediction of commercial strains, and identified 3 of the 30 phenotypic tests (growth in musts containing iprodion (0.05 mg/mL), cycloheximide (0.1 *µ*g/mL) or potassium bisulphite (150 mg/mL)) as the ones providing more information for the assignment of strains to the commercial group. When using only 3 tests, rather than the entire phenotypic profile, the probability of a strain to be classified as commercial increases significantly (from 27% to 95%).

In conclusion, our results demonstrate the usefulness of computational approaches to describe phenotypic variability among groups of *S. cerevisiae* strains that also might occur as adaptive mechanisms in specific environments. The herein developed models can make predictions about the biotechnological potential of strains and simplify the selection of candidate strains to be used as commercial wine strains.

## Materials and Methods

### Strain collection

A *Saccharomyces cerevisiae* strain collection was constituted, comprising 172 strains with different geographical origins and technological applications or origins (Figure 1 and Table S1 – supplementary data). This collection includes strains used for winemaking (commercial and natural isolates that were obtained from winemaking environments), brewing, bakery, distillery (sake, cachaça) and ethanol production, laboratory strains and also strains from particular environments (e.g. pathogenic strains, isolates from fruits, soil and oak exudates). The complete genome sequence of thirty strains is currently available [12] (their original strain code is mentioned in the map of Figure 1). All strains were coded (Zn) and stored at −80°C in cryotubes containing 1 mL glycerol (30% v/v).

### Phenotypic characterization

Phenotypic screening was performed considering a wide range of physiological traits that are also important from an oenological point of view.

In a first set of phenotypic tests, strains were inoculated into replicate wells of 96-well microplates. Isolates were grown overnight in YPD medium (yeast extract 1% w/v, peptone 1% w/v, glucose 2% w/v), and the optical density (A_640_) was then determined and adjusted to 1.0. After washing with peptone (1% w/v), 15 *μ*L of this suspension were inoculated in quadruplicate in microplate wells containing 135 *μ*L of white grape must of the variety Loureiro, to a cellular density of 5×10^6^ cells/mL (A_640_  = 0.1). Final optical density was determined after 22 h (30°C, 200 rpm) in a microplate spectrophotometer. All microplates were carefully sealed with parafilm, and no evaporation was observed for incubation temperatures of 30°C and 40°C. As shown in Table 1, this approach included the following tests: growth at various temperatures (18, 30 and 40°C), evaluation of ethanol resistance (6, 10 and 14%, v/v), tolerance to several stress conditions caused by extreme pH values (2 and 8), osmotic/saline stress (0.75 M KCl and 1.5 M NaCl). Growth was also assessed in the presence of potassium bisulfite (KHSO_3_, 150 and 300 mg/L), copper sulphate (CuSO_4_, 5 mM), sodium dodecyl sulphate (SDS, 0.01%, w/v), the fungicides iprodion (0.05 and 0.1 mg/mL) and procymidon (0.05 and 0.1 mg/mL), as well as cycloheximide (0.05 and 0.1 mg/mL). These tests were carried out using Loureiro grape must supplemented with the mentioned compounds. The growth in finished wines was determined by adding glucose (0.5 and 1%, w/v) to a commercial white wine (12.5% v/v alcohol content). Galactosidase activity was evaluated by adding galactose (5% w/v) to Yeast Nitrogen Base (YNB, Difco^TM^, Ref. 239210), using test tubes with 5 mL culture medium and 5×10^6^ cells/mL, followed by 5 to 6 days of incubation at 26°C.

Other tests were performed using solid media. Overnight cultures were prepared as previously described, adjusted to an optical density (A_640_) of 10.0 and washed. One *µ*l of this suspension was placed on the surface of the culture media mentioned below. Hydrogen sulphide production was evaluated using BiGGY medium (SIGMA-ALDRICH, Ref. 73608) [36], followed by incubation at 27°C for 3 days. The colony colour, which represents the amount of H_2_S produced was then analysed, attributing a score from 0 (no colour change) to 3 (dark brown colony). Ethanol resistance (12%, v/v) and the combined resistance to ethanol (12, 14, 16 and 18%, v/v) and sodium bisulphite (Na_2_S_2_O_5;_ 75 and 100 mg/L) was evaluated by adding the mentioned compounds to Malt Extract Agar (MEA, SIGMA-ALDRICH, Ref. 38954), and growth was visually scored after incubation (2 days at 27°C).

All phenotypic results were assigned to a class between 0 and 3 (0: no growth (A_640_  = 0.1) or no visible growth on solid media or no colour change of the BiGGY medium; 3: at least 1.5 fold increase of A_640,_ extensive growth on solid media or a dark brown colony formed in the BiGGY medium; scores 1 and 2 corresponded to the respective intermediate values) as shown in table S2.

### Data analysis

The phenotypic variability was evaluated by principal component analysis (PCA), available in the Unscrambler X software (Camo). The BioNumerics software (Applied Maths) was used for clustering, dendogram drawing and calculation of cophenetic correlation coefficients. Mann-Whitney test was applied to the phenotypic data set, including Bonferroni correction, to find relevant associations between phenotypic data and the strain's technological or geographical origin. A set of standard predictive data-mining methods, such as naïve Bayesian classifier and *k* nearest-neighbours algorithm [21], as implemented in the Orange data mining suite [37], [38], were used for the inference of prediction models. For prediction scoring, area under the receiver operating characteristics (ROC) curve (AUC) was used [22], which estimates the probability that the predictive model would correctly differentiate between distinct locations or distinct technological application or origins, given the associated pairs of strains.

## Supporting Information

Figure S1 **Phenotypic variation of 172 strains under 30 growth conditions.** Strains are organized according to UPGMA-based hierarchical clustering (cophenetic correlation factor  = 0.75), using Euclidean distance correlation to estimate phenotypic profile similarities. Symbols represents the strains technological applications or origin: black star – wine and vine; grey star – commercial wine strain; black square – clinical; grey square – natural isolates; black circle – sake; grey circle – other fermented beverages; black pentagon – beer; grey pentagon- baker; black rectangle – laboratory; grey rectangle – unknown biological origin.
(TIF)

Figure S2 **PCA representation of the three strain clusters, obtained with** ***k*** **-means clustering algorithm.** The symbols represent the belonging of the 172 strains shown in the phenotypic data PCA (Figure 2b) to each cluster: circles – cluster 1 (38 strains); lines – cluster 2 (90 strains); squares – cluster 3 (44 strains).
(TIF)

Table S1 **Origin and technological application of the 172** ***Saccharomyces cerevisiae*** **strains.**
(DOCX)

Table S2 (XLSX)

## References

[1] Fleet GH (1998) Yeasts – What reactions and interactions really occur in natural habitats. Food Technol. Biotechnol. 36: 285–289.

[2] Schuller D (2010) Better yeast for better wine – genetic improvement of *Saccharomyces cerevisiae* wine strains. In: Rai M, Koevics G, editors. Progress in mycology. Jodhpur: Scientific Publishers (India). 1–51.

[3] Camarasa C, Sanchez I, Brial P, Bigey F, Dequin S (2011) Phenotypic landscape of *Saccharomyces cerevisiae* during wine fermentation: Evidence for origin-dependent metabolic traits. PloS one 6: e25147. 21949874 10.1371/journal.pone.0025147 PMC3174997

[4] Bisson LF (1999) Stuck and sluggish fermentations. Am J Enol Vitic 50: 107–119.

[5] Frezier V, Dubourdieu D (1992) Ecology of yeast strains *Saccharomyces cerevisiae* during spontaneous fermentation in Bordeaux winery. Am J Enol Vitic 43: 375–380.

[6] Lopes CA, Broock M Van, Querol A, Caballero AC (2002) *Saccharomyces cerevisiae* wine yeast populations in a cold region in Argentinean Patagonia. A study at different fermentation scales. J Appl Microbiol 93: 608–615. 12234344 10.1046/j.1365-2672.2002.01738.x

[7] Sabate J, Cano J, Querol A, Guillamo JM (1998) Diversity of *Saccharomyces* strains in wine fermentations: analysis for two consecutive years. Lett Appl Microbiol 26: 452–455. 9717318 10.1046/j.1472-765x.1998.00369.x

[8] Schuller D, Alves H, Dequin S, Casal M (2005) Ecological survey of *Saccharomyces cerevisiae* strains from vineyards in the Vinho Verde Region of Portugal. FEMS Microbiol Ecol 51: 167–177. 16329865 10.1016/j.femsec.2004.08.003

[9] Valero E, Cambon B, Schuller D, Casal M, Dequin S (2007) Biodiversity of *Saccharomyces* yeast strains from grape berries of wine-producing areas using starter commercial yeasts. FEMS Yeast Res 7: 317–329. 17040482 10.1111/j.1567-1364.2006.00161.x

[10] Kvitek DJ, Will JL, Gasch AP (2008) Variations in stress sensitivity and genomic expression in diverse *Saccharomyces cerevisiae* isolates. PLoS Genet 4: 31–35. 10.1371/journal.pgen.1000223 PMC2562515 18927628

[11] Greig D, Leu JY (2009) Natural history of budding yeast. Curr Biol 19: 886–890. 10.1016/j.cub.2009.07.037 19825346

[12] Liti G, Carter DM, Moses AM, Warringer J, Parts L, et al. (2009) Population genomics of domestic and wild yeasts. Nature 458: 337–341. Available: http://www.pubmedcentral.nih.gov/articlerender.fcgi?artid=2659681&tool=pmcentrez&rendertype=abstract. Accessed 2 March 2012. 10.1038/nature07743 PMC2659681 19212322

[13] Schacherer J, Shapiro JA, Ruderfer DM, Kruglyak L (2009) Comprehensive polymorphism survey elucidates population structure of *Saccharomyces cerevisiae* . Nature 458: 342–345. 19212320 10.1038/nature07670 PMC2782482

[14] Fay JC, Benavides J (2005) Evidence for domesticated and wild populations of *Saccharomyces cerevisiae* . PLoS Genet 1: 66–71. 16103919 10.1371/journal.pgen.0010005 PMC1183524

[15] Briones AI, Ubeda JF, Cabezudo MD, Martin-Alvarez P (1995) Selection of spontaneous strains of Saccharomyces cerevisiae as starters in their viticultural area. In: Charalambous G, editor. Food flavours: generation, analysis and process influence. Amsterdam: Elsevier Science. 1597–1622.

[16] Ramirez M, Perez F, Regodon JA (1998) A simple and reliable method for hybridization of homothallic wine strains of *Saccharomyces cerevisiae* . Appl Environ Microbiol 64: 5039–5041. 9835605 10.1128/aem.64.12.5039-5041.1998 PMC90965

[17] Mannazzu I, Clementi F, Ciani M (2002) Strategies and criteria for the isolation and selection of autochthonous starter. In: Ciani M, editor. Biodiversity and biotechnology of wine yeasts. Trivandrum: Research Signpost. 19–35.

[18] Franco-Duarte R, Umek L, Zupan B, Schuller D (2009) Computational approaches for the genetic and phenotypic characterization of a *Saccharomyces cerevisiae* wine yeast collection. Yeast 26: 675–692. 19894212 10.1002/yea.1728

[19] Rousseeuw PJ (1987) Silhouettes: A graphical aid to the interpretation and validation of cluster analysis. Journal of Computational and Applied Mathematics 20: 53–65 Available: http://linkinghub.elsevier.com/retrieve/pii/0377042787901257.

[20] Grimshaw SD, Efron B, Tibshirani RJ (1995) An Introduction to the Bootstrap. Technometrics 37: 341.

[21] Tan P, Steinbach M, Kumar V (2006) Introduction to data mining. Pearson Ed. Boston: Pearson Addison Wesley.

[22] Hanley JA, McNeil BJ (1982) The meaning and use of the area under a receiver operating characteristic (ROC) curve. Radiology 143: 29–36. 7063747 10.1148/radiology.143.1.7063747

[23] Mozina M, Demsar J, Kattan M, Zupan B (2004) Nomograms for visualization of naive Bayesian classifier. Lecture Notes in Computer Science 3202: 337–348.

[24] Agnolucci M, Scarano S, Santoro S, Sassano C, Toffanin A, et al (2007) Genetic and phenotypic diversity of autochthonous Saccharomyces spp. strains associated to natural fermentation of “Malvasia delle Lipari”. Lett Appl Microbiol 45: 657–662. 17922817 10.1111/j.1472-765X.2007.02244.x

[25] Brandolini V, Tedeschi P, Capece A, Maietti A, Mazzotta D, et al (2002) *Saccharomyces cerevisiae* wine strains differing in copper resistance exhibit different capability to reduce copper content in wine. World J Microbiol Biotechnol 18: 499–503.

[26] Salinas F, Mandakovic D, Urzua U, Massera A, Miras S, et al (2010) Genomic and phenotypic comparison between similar wine yeast strains of *Saccharomyces cerevisiae* from different geographic origins. J Appl Microbiol 108: 1850–1858. 20163487 10.1111/j.1365-2672.2010.04689.x

[27] Cubillos F a, Zia A, Gjuvsland A, Jared T, Warringer J, et al. (2011) Trait variation in yeast is defined by population history. PLoS Genet 7: e1002111. Available: http://www.pubmedcentral.nih.gov/articlerender.fcgi?artid=3116910&tool=pmcentrez&rendertype=abstract. Accessed 8 November 2012. 10.1371/journal.pgen.1002111 PMC3116910 21698134

[28] Schacherer J, Ruderfer DM, Gresham D, Dolinski K, Botstein D, et al (2007) Genome-wide analysis of nucleotide-level variation in commonly used *Saccharomyces cerevisiae* strains. PLoS One 2: e322. 17389913 10.1371/journal.pone.0000322 PMC1829191

[29] Goddard MR, Anfang N, Tang R, Gardner RC, Jun C (2010) A distinct population of Saccharomyces cerevisiae in New Zealand: evidence for local dispersal by insects and human-aided global dispersal in oak barrels. Environ Microbiol 12: 63–73 Available: http://www.ncbi.nlm.nih.gov/entrez/query.fcgi?cmd=Retrieve&db=PubMed&dopt=Citation&list_uids=19691498. 19691498 10.1111/j.1462-2920.2009.02035.x

[30] Muller LH, McCusker JH (2009) Microsatellite analysis of genetic diversity among clinical and nonclinical Saccharomyces cerevisiae isolates suggests heterozygote advantage in clinical environments. Mol Ecol 18: 2779–2786. 19457175 10.1111/j.1365-294X.2009.04234.x PMC2768266

[31] Schuller D, Pereira L, Alves H, Cambon B, Dequin S, et al (2007) Genetic characterization of commercial *Saccharomyces cerevisiae* isolates recovered from vineyard environments. Yeast 24: 625–636. 17534867 10.1002/yea.1496

[32] Dunn B, Levine RP, Sherlock G (2005) Microarray karyotyping of commercial wine yeast strains reveals shared, as well as unique, genomic signatures. BMC genomics 6: 53 doi:10.1186/1471-2164-6-53 15833139 10.1186/1471-2164-6-53 PMC1097725

[33] Legras J-L, Merdinoglu D, Cornuet J-M, Karst F (2007) Bread, beer and wine: *Saccharomyces cerevisiae* diversity reflects human history. Mol Ecol 16: 2091–2102. 17498234 10.1111/j.1365-294X.2007.03266.x

[34] Chiai NO, Ujimura MF, Shima MO, Otoyama TM, Chiishi AI, et al (2002) Efects of iprodione and fludioxonil on glycerol synthesis and hyphal development in Candida albicans. Biosci Biotechnol Biochem 66: 2209–2215. 12450134 10.1271/bbb.66.2209

[35] Cadez N, Zupan J, Raspor P (2010) The effect of fungicides on yeast communities associated with grape berries. FEMS Yeast Res 10: 619–630. 20491940 10.1111/j.1567-1364.2010.00635.x

[36] Jiranek V, Langridge P, Henschke PA (1995) Validation of bismuth-containing indicator media for predicting H2S-producing potential of Saccharomyces cerevisiae wine yeasts under enological conditions. Am J Enol Vitic 46: 269–273.

[37] Curk T, Demsar J, Xu Q, Leban G, Petrovic U, et al (2005) Microarray data mining with visual programming. Bioinformatics 21: 396–398. 15308546 10.1093/bioinformatics/bth474

[38] Demsar J, Zupan B, Leban G (2004) Orange: from experimental machine learning to interactive data mining. White Paper (www.ailab.si/orange), Faculty of Computer and Information Science, University of Ljubljana.

