# Supplementary figures and images for: Computational Models for Prediction of Yeast Strain Potential for Winemaking from Phenotypic Profiles
Source: PLoS One. 2013 Jul 16;8(7):e66523. doi: 10.1371/journal.pone.0066523 (PMC3713011; doi:10.1371/journal.pone.0066523)

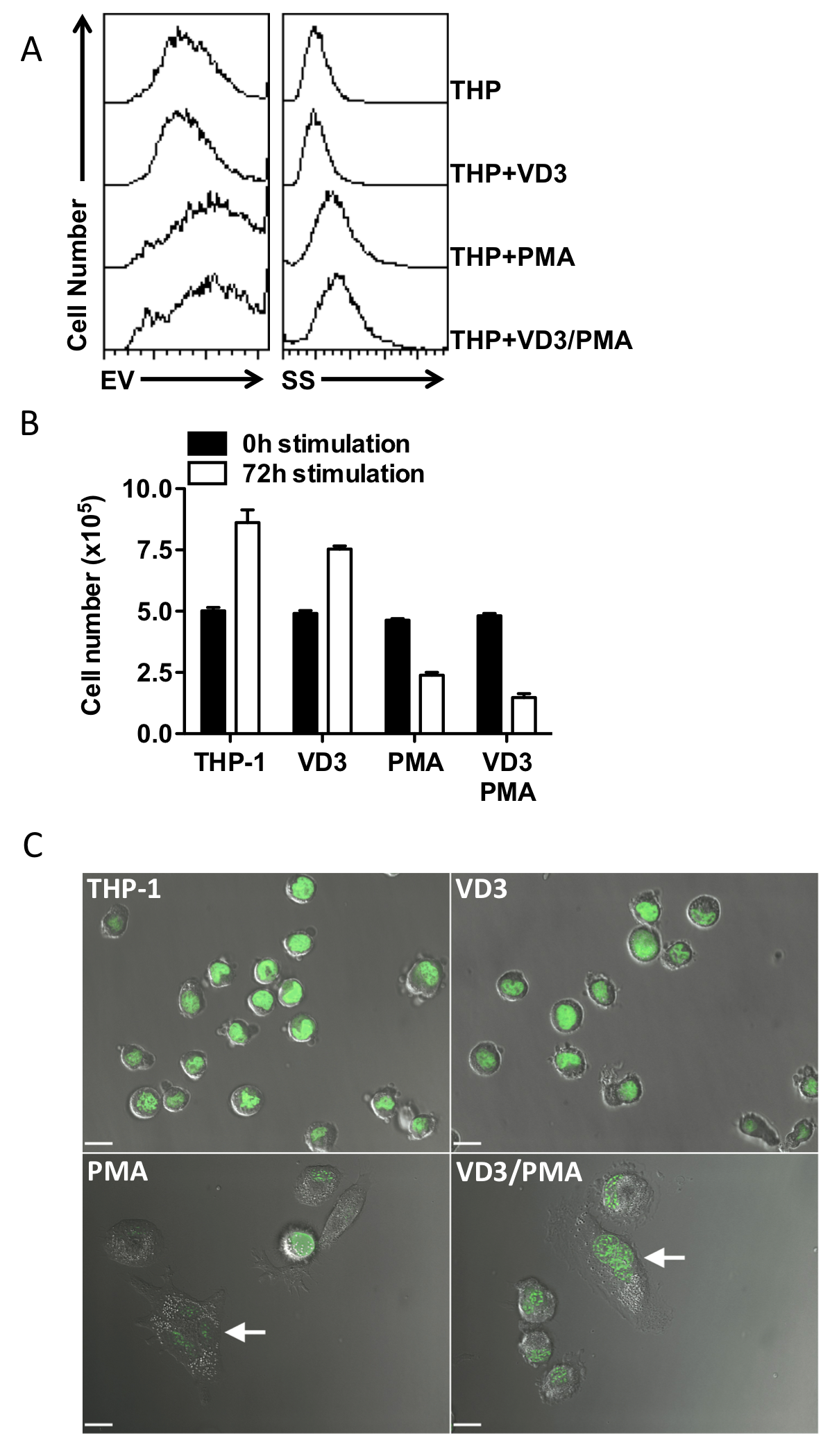

Supplement: Figure S1 — Phenotypic variation of 172 strains under 30 growth conditions. Strains are organized according to UPGMA-based hierarchical clustering (cophenetic correlation factor = 0.75), using Euclidean distance correlation to estimate phenotypic profile similarities. Symbols represents the strains technological applications or origin: black star – wine and vine; grey star – commercial wine strain; black square – clinical; grey square – natural isolates; black circle – sake; grey circle – other fermented beverages; black pentagon – beer; grey pentagon- baker; black rectangle – laboratory; grey rectangle – unknown biological origin. (TIF) [file pone.0066523.s001.tif]

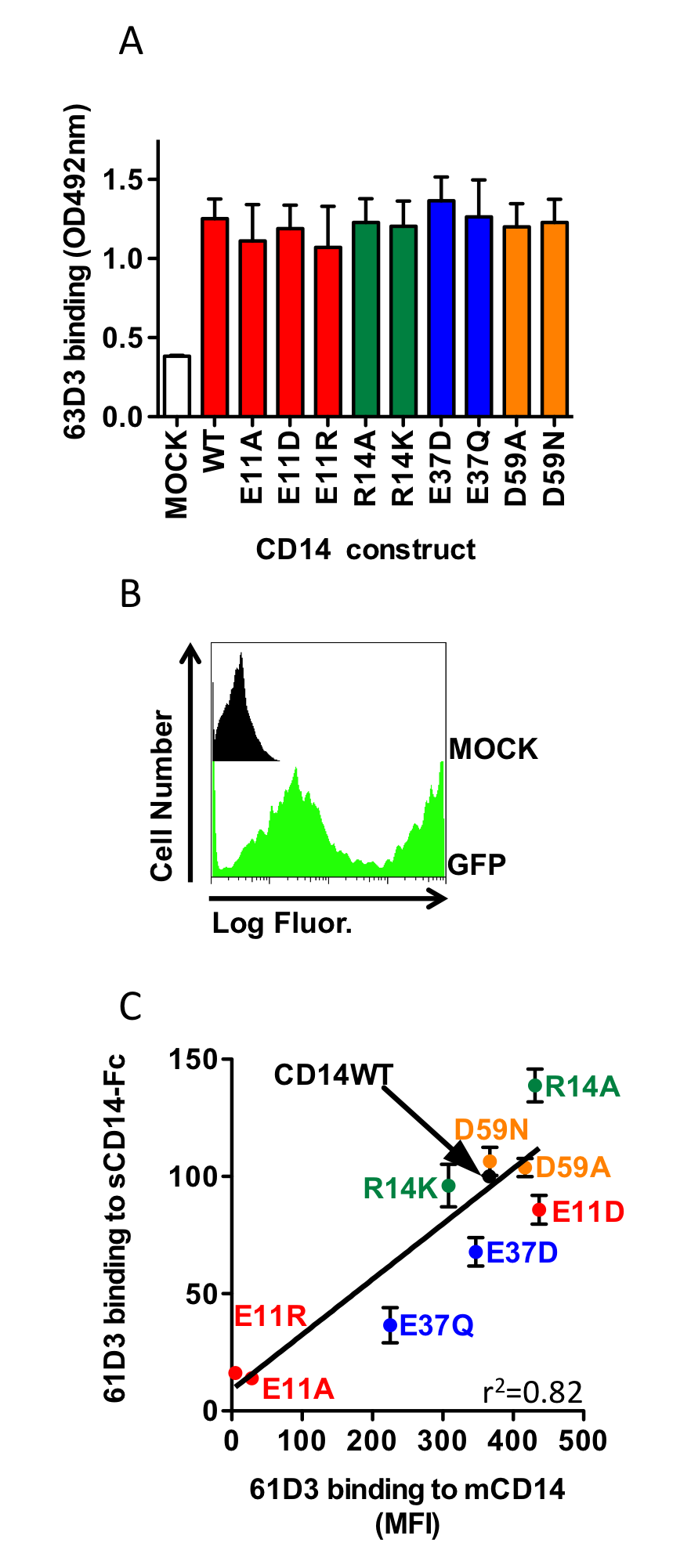

Supplement: Figure S2 — PCA representation of the three strain clusters, obtained with k -means clustering algorithm. The symbols represent the belonging of the 172 strains shown in the phenotypic data PCA (Figure 2b) to each cluster: circles – cluster 1 (38 strains); lines – cluster 2 (90 strains); squares – cluster 3 (44 strains). (TIF) [file pone.0066523.s002.tif]
