# Supplementary material for: Computational Models for Prediction of Yeast Strain Potential for Winemaking from Phenotypic Profiles
Source: PLoS One. 2013 Jul 16;8(7):e66523. doi: 10.1371/journal.pone.0066523 (PMC3713011; doi:10.1371/journal.pone.0066523)
Supplement: Table S1 — Origin and technological application of the 172 Saccharomyces cerevisiae strains. (DOCX) [file pone.0066523.s003.docx]

**Table S1.** Origin and technological application of the 172 *Saccharomyces cerevisiae* strains

| **Number of isolates** | **Geographical Origin** | **Technological application** | **Provided by** | **Liti´s original designation [12]** |
| --- | --- | --- | --- | --- |
| 1 | France | Laboratory | Liti, G. | 97 Y55 |
| 1 | USA | Laboratory | Liti, G. | 17 SK1 |
| 1 | Italy | Clinical | Liti, G. | 303 YJM978 |
| 1 | Italy | Clinical | Liti, G. | 304 YJM981 |
| 1 | Italy | Clinical | Liti, G. | 308 YJM975 |
| 1 | UK | Clinical | Liti, G. | 284 322134S |
| 1 | UK | Clinical | Liti, G. | 287 378604X |
| 1 | UK | Clinical | Liti, G. | 288 273614N |
| 1 | Finland | Natural isolate | Liti, G. | 84 DBVPG1788 |
| 1 | Netherlands | Natural isolate | Liti, G. | 91 DBVPG1373 |
| 1 | France | Commercial wine strain | Liti, G. | 174 YIIc17_E5 |
| 1 | Netherlands | Other fermented beverages | Liti, G. | 155 DBVPG6040 |
| 1 | Ireland | Beer | Liti, G. | 248 NCYC361 |
| 1 | USA | Natural isolate | Liti, G. | 182 YPS606 |
| 1 | USA | Natural isolate | Liti, G. | 104 YPS128 |
| 1 | Australia | Bread | Liti, G. | 258 YS2 |
| 1 | Netherlands | Bread | Liti, G. | 259 YS4 |
| 1 | Singapore | Bread | Liti, G. | 262 YS9 |
| 1 | USA | Wine and vine | Liti, G. | 181 BC187 |
| 1 | Malaysia | Natural isolate | Liti, G. | 278 UWOPS03-461.4 |
| 1 | Malaysia | Natural isolate | Liti, G. | 279 UWOPS05-217.3 |
| 1 | Malaysia | Natural isolate | Liti, G. | 280 UWOPS05-227.2 |
| 1 | Japan | Saké | Liti, G. | 251 K11 |
| 1 | Indonesia | Saké | Liti, G. | 252 Y9 |
| 1 | USA | Wine and vine | Liti, G. | 345 RM11 |
| 1 | Ethiopia | Bread | Liti, G. | 92 DBVPG1853 |
| 1 | Ivory Coast | Other fermented beverages | Liti, G. | 253 Y12 |
| 1 | West Africa | Other fermented beverages | Liti, G. | 247 NCYC110 |
| 1 | West Africa | Other fermented beverages | Liti, G. | 60 DBVPG6044 |
| 1 | Unknown | Unknown | Liti, G. | 3 DBVPG6765 |
| 1 | Portugal | Unknown biological origin | Liti, G. | OV 382 |
| 1 | Chile | Wine and vine | Liti, G. | 220 L-1374 |
| 1 | Chile | Wine and vine | Liti, G. | 221 L-1528 |
| 1 | Hawaii | Natural isolate | Liti, G. | 271 UWOPS87-2421 |
| 1 | Australia | Natural isolate | Liti, G. | 150 DBVPG1106 |
| 1 | Bahamas | Natural isolate | Liti, G. | 270 UWOPS83-787.3 |
| 3 | Portugal | Clinical | Carreto L. |  |
| 4 | Portugal | Wine and vine | Carreto L. |  |
| 4 | Japan | Saké | Goto-Yakamoto, N. |  |
| 1 | Unknown | Natural isolate | Kurtzman, C.P. |  |
| 1 | Africa | Other fermented beverages | Kurtzman, C.P. |  |
| 1 | Indonesia | Natural isolate | Kurtzman, C.P. |  |
| 1 | West Africa | Other fermented beverages | Kurtzman, C.P. |  |
| 1 | French Guiana | Unknown | Kurtzman, C.P. |  |
| 1 | Turkey | Wine and vide | Kurtzman, C.P. |  |
| 1 | Indonesia | Other fermented beverages | Kurtzman, C.P. |  |
| 1 | Philippines | Other fermented beverages | Kurtzman, C.P. |  |
| 1 | Ivory Coast | Other fermented beverages | Kurtzman, C.P. |  |
| 3 | Brazil | Other fermented beverages | Brandão, R. |  |
| 8 | Turkey | Wine and vine | Huseyin, E. |  |
| 13 | France | Wine and vine |  |  |
| 1 | Unknown | Laboratory |  |  |
| 1 | Unknown | Unknown |  |  |
| 30 | France | Commercial wine strain |  |  |
| 2 | Germany | Commercial wine strain |  |  |
| 3 | Portugal | Commercial wine strain |  |  |
| 2 | South Africa | Commercial wine strain |  |  |
| 1 | Spain | Commercial wine strain |  |  |
| 1 | USA | Commercial wine strain |  |  |
| 7 | Unknown | Commercial wine strain |  |  |
| 44 | Portugal | Wine and vine |  |  |
